# Minimum information and guidelines for reporting a multiplexed assay of variant effect

**DOI:** 10.1186/s13059-024-03223-9

**Published:** 2024-04-19

**Authors:** Melina Claussnitzer, Victoria N. Parikh, Alex H. Wagner, Jeremy A. Arbesfeld, Carol J. Bult, Helen V. Firth, Lara A. Muffley, Alex N. Nguyen Ba, Kevin Riehle, Frederick P. Roth, Daniel Tabet, Benedetta Bolognesi, Andrew M. Glazer, Alan F. Rubin

**Affiliations:** 1 The Novo Nordisk Foundation Center for Genomic Mechanisms of Disease, Broad Institute of MIT and Harvard, Cambridge, MA 02142 USA; 2 Center for Genomic Medicine, Massachusetts General Hospital, Harvard Medical School, Cambridge, MA 02142 USA; 3 Stanford Center for Inherited Cardiovascular Disease, Stanford University School of Medicine, Stanford, CA 94305 USA; 4 The Steve and Cindy Rasmussen Institute for Genomic Medicine, Nationwide Children’s Hospital, Columbus, OH 43215 USA; 5 Department of Pediatrics, The Ohio State University College of Medicine, Columbus, OH 43210 USA; 6 Department of Biomedical Informatics, The Ohio State University, Columbus, OH 43210 USA; 7 The Jackson Laboratory, Bar Harbor, ME 04609 USA; 8 Wellcome Sanger Institute, Hinxton, Cambridge, UK; 9 Dept of Medical Genetics, Cambridge University Hospitals NHS Trust, Cambridge, UK; 10 Department of Genome Sciences, University of Washington, Seattle, WA 98105 USA; 11 Department of Biology, University of Toronto at Mississauga, Mississauga, ON Canada; 12 Department of Molecular and Human Genetics, Baylor College of Medicine, Houston, TX 77030 USA; 13 Donnelly Centre, University of Toronto, Toronto, ON Canada; 14 Department of Molecular Genetics, University of Toronto, Toronto, ON Canada; 15 Department of Computer Science, University of Toronto, Toronto, ON Canada; 16 Lunenfeld-Tanenbaum Research Institute, Sinai Health, Toronto, ON Canada; 17 Institute for Bioengineering of Catalunya (IBEC), The Barcelona Institute of Science and Technology, Barcelona, Spain; 18 Vanderbilt University Medical Center, Nashville, TN 37232 USA; 19 Bioinformatics Division, The Walter and Eliza Hall Institute of Medical Research, Parkville, VIC Australia; 20 Department of Medical Biology, University of Melbourne, Parkville, VIC Australia

**Keywords:** Genomics, Standards, Genetic variants, Multiplexed assays of variant effect, MAVE, Deep mutational scanning, DMS

## Abstract

Multiplexed assays of variant effect (MAVEs) have emerged as a powerful approach for interrogating thousands of genetic variants in a single experiment. The flexibility and widespread adoption of these techniques across diverse disciplines have led to a heterogeneous mix of data formats and descriptions, which complicates the downstream use of the resulting datasets. To address these issues and promote reproducibility and reuse of MAVE data, we define a set of minimum information standards for MAVE data and metadata and outline a controlled vocabulary aligned with established biomedical ontologies for describing these experimental designs.

**Supplementary Information:**

The online version contains supplementary material available at 10.1186/s13059-024-03223-9.

## Background

The emergence of high-throughput genomic technologies has revolutionized our ability to study the impact of genetic variants at a grand scale. A prominent example of these innovative methods is multiplexed assays of variant effect (MAVEs). MAVEs are a family of experimental methods combining saturation mutagenesis with a multiplexed assay to interrogate the effects of thousands of genetic variants in a given functional element in parallel [1, 2]. The output of a MAVE is a variant effect map quantifying the consequences of all single nucleotide (or single amino acid) variants in a target functional element, even variants not yet observed in the population. MAVEs have been applied to coding sequences as well as noncoding elements like splice sites and regulatory regions across various organisms. Variant effect maps have broad applications including clinical variant interpretation [2, 3], understanding sequence/structure/function relationships [4, 5], and investigating molecular mechanisms of evolution [6, 7]. The MAVE field is growing rapidly, leading to the formation of organizations such as the Atlas of Variant Effects (AVE). AVE consists of over 500 researchers from over 30 countries who perform, interpret, and apply MAVE experiments.

The rapid growth and adoption of MAVE technologies across many fields have led to an excess of overlapping definitions, complicating discovery and interpretation. Minimum information standards in other research areas have increased the reporting, archiving, and reuse of biological data [8–11]. To promote reuse and FAIR data sharing [12], minimum information standards and a controlled vocabulary for describing MAVE experiments and variant effect maps are needed. Here, we—members of the AVE Experimental Technology and Standards and AVE Data Coordination and Dissemination workstreams—provide a comprehensive structured vocabulary and recommendations for data release for MAVE datasets. Uptake of these recommendations by the MAVE community will greatly improve the usability and longevity of MAVE datasets, enabling novel insights and applications.

## Results and discussion

**Fig. 1:**
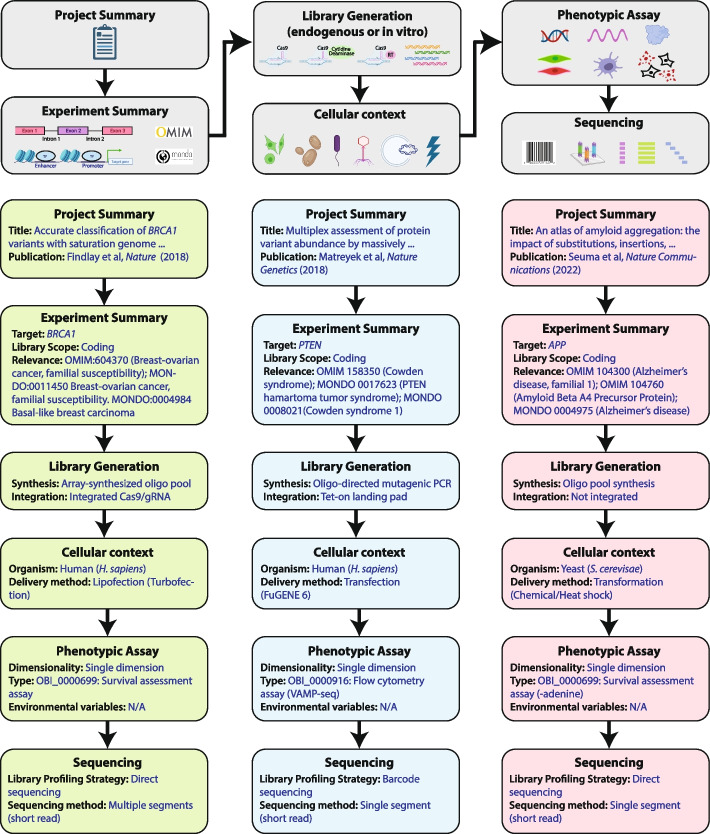
A structured vocabulary of terms relevant to the technical development, execution and recording of multiplexed assays of variant effect (MAVEs). Each category of controlled vocabulary terms is depicted (top, gray boxes) along with three examples from published MAVE datasets. From left to right, the figure includes (green boxes) [17], (blue boxes) [18], and (red boxes) [19]. Example files for each of these examples are available in the GitHub repository (see Availability of data and materials)

All MAVEs share a core pipeline: generation of a variant library, delivery of the library into a model system, separation of variants based on function, quantification of variant frequency by high-throughput DNA sequencing, and performing of data analysis and score calculation [1, 2, 13]. Accurate and consistent metadata describing each of these steps is the basis for the interpretability of MAVE functional scores and is a requirement for any advanced quantitative analysis, such as comparing and combining scores. To systematize these metadata, we have defined and implemented a computable controlled vocabulary that covers the majority of current and emerging MAVE techniques (Fig. 1) [14]. This vocabulary captures the major steps of the MAVE experimental process including project scope, library generation, library integration/expression, assay type, and sequencing method. The vocabulary also contains terms to describe the biological and disease relevance of the assay. In addition to releasing scores and other datasets in published papers, we recommend sharing MAVE datasets through MaveDB, an open-source platform to distribute and interpret MAVE data [15, 16].

Researchers should communicate the target sequence, the method used to generate library diversity, and the method of variant delivery into the assay system using terms from the controlled vocabulary. Metadata about the variant generation method should include terms for either editing at the endogenous locus or in vitro variant library generation. It should also specify the model system as defined by NCBI Taxonomy ID [20] and Cell Line Ontology (CLO) [21] terms where available.

It is essential for the target sequence to be linked to a reference genome database or similar by including a versioned stable identifier from a widely used resource such as RefSeq [22], Ensembl [23], or UniProt [24]. We also recommend that researchers designing a study choose a reference-identical allele when it does not otherwise affect the study design, particularly for clinically relevant targets. The entire target sequence used in the assay must be provided to allow MaveDB and other systems to generate globally unique identifiers (sha512t24u computed identifiers [25]) as used by the Global Alliance for Genomics and Health (GA4GH) [26] refget [27] and Variation Representation Specification (VRS) [28] standards.

We recommend that variant libraries are exchanged using VRS and stored using a VRS-compatible information model, including the aforementioned computed identifiers, inter-residue sequence location data, and VOCA-normalized allele representation [28, 29]. This allows variants to be defined in terms of both the variant on the target sequence and the homologous variant on the linked reference sequences with an appropriate variant mapping relation, such as the *homologous_to* relation from the sequence ontology [30]. Descriptions of variants on target sequences should follow the MAVE-HGVS nomenclature conventions [16]. Homologous variants on linked reference sequences should describe variants following conventions typical for the target organism, e.g., using the Human Genome Variation Society (HGVS) variant nomenclature [31] for variants on human reference sequences. An example of these sequence variant recommendations in practice is described in Arbesfeld et al. [32], where they enable interoperability with downstream resources including the Ensembl Variant Effect Predictor (VEP) [33], UCSC Genome Browser [34], the Genomics to Proteins resource [35], the ClinGen Allele Registry [36], and ClinGen Linked Data Hub.

The phenotypic assay is the most unique aspect of a MAVE compared to other data types for which minimum information standards have been established. There is a tremendous diversity in functional assays in terms of both the assay readout and the biology the assay was designed to interrogate. For assay readout, we have identified a subset of phenotypic readouts in the Ontology for Biomedical Investigations (OBI) [37] that are commonly used in variant effect maps. Because OBI has over 2500 terms, we hope that this “short list” will help researchers identify the most relevant terms to describe their experiments. Nevertheless, we also welcome the use of other OBI terms if necessary to describe new assays. Assays that used variants with known effects to calibrate or validate the assay should include these variants, their effects, and the source of the information [38]. To promote interoperability, we suggest using a structured format such as a table or JSON document and applying the VRS standard as described above. Researchers should also detail any environmental variables (such as temperature or the addition of small molecules) in their experimental methods. We encourage experimenters to use publicly accessible resources like protocols.io to describe their assay protocols in detail and share them with the community.

Researchers should use the appropriate controlled vocabulary terms for describing the high-throughput sequencing method used for variant quantification. We strongly recommend that raw sequence reads be deposited in a suitable repository, such as the Sequence Read Archive (SRA) [39] or Gene Expression Omnibus (GEO) [40], along with a description of each file (e.g., time point and sample information).

We recommend that researchers investigating clinical phenotypes use terms from the Mondo Disease Ontology (Mondo) [41] or Online Mendelian Inheritance in Man (OMIM) [42] to help clinicians and other stakeholders retrieve relevant functional data. Particular care is needed for genes encoding proteins with multiple functional domains and where loss of function and gain of function variants are associated with different diseases. Ideally, each MAVE should be associated with a particular gene-disease entity that describes the mechanism of disease such as those defined by G2P [43] and how the MAVE assay recapitulates or is relevant to the mechanism of disease. Some genes or functional domains may require multiple MAVE assays, each probing a different function or attribute of the gene product, to accurately model different disease entities.

Although it is not within the scope of this controlled vocabulary, it is still crucial to detail the data analysis performed to produce a variant effect map. This includes steps to generate variant counts, including sequence read processing, quality filtering, alignment, and variant identification, as well as further statistical and bioinformatic processing to calculate scores and associated error estimates. Researchers should describe the analysis pipeline used for these calculations, including software versions. Several well-documented tools are available for this purpose and the field continues to advance rapidly [44–47]. Custom code should be shared using GitHub or a similar platform and archived using Zenodo or a similar archival service that mints a DOI.

In addition to processed variant scores, we urge researchers to share raw counts for each dataset, as these have tremendous utility for downstream users who want to reanalyze datasets or develop new statistical methods. Similarly, we recommend that researchers also report scores prior to normalization or imputation, and MaveDB supports the deposition of counts, scores, normalized/imputed scores, and sequence metadata for the same dataset (Table 1).

**Table 1:** Recommended locations for MAVE data deposition

**Type of data**	**Deposition location**
Processed scores, unprocessed scores, raw counts	MaveDB [15, 16]
Raw sequence reads	Sequence Read Archive [39]/Gene Expression Omnibus [40]
Target sequence	MaveDB [15, 16]
Linked sequence references	MaveDB [15, 16]
Sequence metadata/digests	MaveDB [15, 16]/SeqRepo [25]
Variant library	MaveDB [15, 16]
Analysis code	GitHub/Zenodo
Structured vocabulary description	This work/MaveDB [15, 16]

## Conclusions

Minimum data standards are important to guide researchers who want their datasets to be used and cited broadly. We anticipate that this document will enhance the readability and discoverability of current and future datasets by defining a vocabulary that can be adopted across the many fields where MAVEs are being performed and where the resulting datasets are being used. Ensuring a minimum set of available metadata that uses a shared set of terms enables new types of analysis, such as machine learning methods to combine large numbers of disparate, high-dimensional datasets like MAVEs. Large-scale meta-analyses of multiple MAVE datasets have already been implemented in several contexts, including computational prediction of variant effects [48, 49] and clinical variant reclassification [50]. In the near term, the controlled vocabulary will be implemented as part of MaveDB records, creating a large set of rich metadata annotations that can be searched and mined. We believe that the MAVE community should share datasets and resources responsibly and that accessibility is real only when it ensures usability and reproducibility.

## Methods

The initial draft of the controlled vocabulary was developed collaboratively using Google Docs. The controlled vocabulary schema is defined using JSON Schema Draft 2020-12.

### Supplementary Information

**Additional file 1.** Review history.

## Data Availability

The controlled vocabulary implementation is available from the AVE Data Coordination and Dissemination workstream GitHub repository located at https://github.com/ave-dcd/mave_vocabulary and stably archived using Zenodo at 10.5281/zenodo.10719897 [14]. The implementation is provided under the Creative Commons Attribution 4.0 International (CC BY 4.0) license.
