# Supplementary material for: Minimum information and guidelines for reporting a multiplexed assay of variant effect
Source: Genome Biol. 2024 Apr 19;25:100. doi: 10.1186/s13059-024-03223-9 (PMC11027375; doi:10.1186/s13059-024-03223-9)
Supplement: Supplementary file 1 — Additional file 1. Review history. [file 13059_2024_3223_MOESM1_ESM.docx]

Review History

**First round of review**

**Reviewer 1**

**Were you able to assess all statistics in the manuscript, including the appropriateness of statistical tests used?**

There are no statistics in the manuscript.

**Comments to author:**

The manuscript describes a minimum information standard for reporting data from Multiplexed Assays of Variant Effect (MAVEs). The contribution is important because a lack of metadata reporting has been a barrier to progress in terms of integrating MAVE data into other databases / resources and in terms of downstream applications of MAVEs such as machine learning and variant interpretation.

The manuscript will be highly cited by other manuscripts in order to indicate compliance with the standard and will be influential in the MAVE community. Genome Biology is an appropriate venue for a paper, such as this one, that establishes key infrastructure for a class of experiments of interest to a wide array of fields (clinical genetics, protein design, gene regulation, infectious disease, cancer, etc.).

I have several suggestions for improvement, with the first 2 being in my view essential for publication.

1. There is a lack of clarity in the manuscript about what exactly the terms in the information standard are and where they are defined. For example, are the gray terms in Figure 1 all of the alternatives for these fields or just a subset of the possibilities?

2. The first issue is exacerbated by the lack of documentation on the github site. I truly could not make heads or tails about how the files in the repository were intended to be used. In order to be widely used, the standard needs to be well-documented, with enough explanatory and didactic information so that an experimentalist with e.g. no more than basic coding experience should be able to follow the instructions to create a valid reporting document.

3. The reporting standard is missing any information at all concerning quality control, experimental design (e.g. technical and biological replicate structure), calibration / validation data availability and methodology, etc.

4. I found the treatment of minimum information guidelines for the phenotypic assay to be insufficient for many downstream purposes. Simply saying that researchers should use the Ontology for Biomedical Investigations vocabulary seems motivated by technical considerations in establishing a controlled vocabulary rather than the equally important goal of encouraging the reporting of the key information needed to ensure the scientific validity of downstream analyses. The authors should consider whether additional information should be specified, such as strain / cell line, media, temperature, number of generations of growth, cell density, etc.. While these values may not be applicable to every one of the broad array of phenotypic assays currently in use, providing examples for the most commonly used classes of experiments (growth, sort-seq, VAMP-seq, etc.) would cover the needs of many users and would also provide helpful general guidance to those attempting to report less commonly used assays.

**Reviewer 2**

**Were you able to assess all statistics in the manuscript, including the appropriateness of statistical tests used?**

There are no statistics in the manuscript.

**Comments to author:**

This study reports the development of minimum information and guidelines for reporting a multiplexed assay of variant effect. Overall, the challenges to be addressed are properly identified, and the method reported is the state-of-the-art.

However, more details appear to be needed for this work. First of all, It is hard to know from current paper what is really "minimal" in terms of the minimal information.

The paper emphasizes the usage of ontologies such as OBI and CLO, which is great. However, more details would be better. For example, it may be possible to provide a full list of ontologies to be used.

it appears difficult to evaluate the quality and value of the minimal information standard. Ideally, the authors need to provide some solid example to demonstrate the usage of the minimal information standard. Currently, Figure 1 provides some simple demo. However, it is hard to evaluate from this example.

**Authors Response**

**Point-by-point responses to the reviewers’ comments:**

We thank the reviewers for their thoughtful and constructive feedback, which helped us improve the manuscript and the associated implementation and documentation. We have replied to each of the reviewers’ comments below in blue.

Reviewer reports:

Reviewer #1: The manuscript describes a minimum information standard for reporting data from Multiplexed Assays of Variant Effect (MAVEs). The contribution is important because a lack of metadata reporting has been a barrier to progress in terms of integrating MAVE data into other databases / resources and in terms of downstream applications of MAVEs such as machine learning and variant interpretation.

The manuscript will be highly cited by other manuscripts in order to indicate compliance with the standard and will be influential in the MAVE community. Genome Biology is an appropriate venue for a paper, such as this one, that establishes key infrastructure for a class of experiments of interest to a wide array of fields (clinical genetics, protein design, gene regulation, infectious disease, cancer, etc.).

I have several suggestions for improvement, with the first 2 being in my view essential for publication.

1. There is a lack of clarity in the manuscript about what exactly the terms in the information standard are and where they are defined. For example, are the gray terms in Figure 1 all of the alternatives for these fields or just a subset of the possibilities?

We agree that the original submission did not do an adequate job of describing the terms in the standard. We have reworked Figure 1 to clarify the difference between categories and examples, as well as adding new examples that we hope will help clarify the use of the vocabulary.

We have also extended the information available on GitHub to include a full list of terms in plain text in the MAVE Vocabulary repository, including a contextual description of terms in the README file (see <https://github.com/ave-dcd/mave_vocabulary/>) and a comprehensive set of terms, definitions and concept mappings in the concept vocabulary .tsv (see <https://github.com/ave-dcd/mave_vocabulary/blob/main/concept_vocabulary.tsv>) , which we hope will help potential users explore all available terms for each category. Because these files are part of the GitHub repository, this content will also be stably archived in Zenodo. We chose to include this information in the repository rather than the main text of the manuscript, since the GitHub content can be more easily updated as the standard continues to evolve.

2. The first issue is exacerbated by the lack of documentation on the github site. I truly could not make heads or tails about how the files in the repository were intended to be used. In order to be widely used, the standard needs to be well-documented, with

enough explanatory and didactic information so that an experimentalist with e.g. no more than basic coding experience should be able to follow the instructions to create a valid reporting document.

We agree and apologize for the lack of documentation that was present initially. We have added more detailed documentation to the GitHub site, including the original example in the figure and additional examples for popular experimental designs that users can use as templates for their own experiments. These examples are also now featured in the revised Figure 1.

We also note that we expect that this standard will be adopted by the community and that more user-friendly tools for generating the schema documents will be developed, but this kind of software implementation is outside the scope for this manuscript, which is intended to define the standard and explain the rationale behind it.

3. The reporting standard is missing any information at all concerning quality control, experimental design (e.g. technical and biological replicate structure), calibration / validation data availability and methodology, etc.

We have added a new section to the schema regarding technical and biological replicate structure, as we agree this is essential information especially for downstream users who want to use the results for modeling. We have also added a new section to the schema where users can describe data availability in a structured format. These new sections were added to all three of the examples provided in the GitHub repository and we hope these will illustrate the use of these sections, as well as the high level of heterogeneity across experiments in the field.

Quality control is very important but it is also a very broad category that we think is best described using a free-text representation in detailed experimental methods, particularly as many QC steps are performed as part of the bioinformatic data analysis and are out of scope for this work.

We have also added text to the manuscript encouraging researchers to provide a list of any control variants used during the assay design or validation, but we think that this information should be provided alongside the minimum information document rather than embedded within it.

4. I found the treatment of minimum information guidelines for the phenotypic assay to be insufficient for many downstream purposes. Simply saying that researchers should use the Ontology for Biomedical Investigations vocabulary seems motivated by technical considerations in establishing a controlled vocabulary rather than the equally important goal of encouraging the reporting of the key information needed to ensure the scientific validity of downstream analyses. The authors should consider whether additional information should be specified, such as strain / cell line, media, temperature, number of generations of growth, cell density, etc.. While these values

may not be applicable to every one of the broad array of phenotypic assays currently in use, providing examples for the most commonly used classes of experiments (growth, sort-seq, VAMP-seq, etc.) would cover the needs of many users and would also provide helpful general guidance to those attempting to report less commonly used assays.

We have clarified the intention behind the minimum information standards and the importance of also sharing detailed protocols. The schema includes optional descriptive text fields that can capture much of this detailed protocol information when important. The AVE Experimental Technology and Standards workstream is actively working to develop a shared protocol repository that will provide detailed information such as cell density and media, and we hope that future studies will take advantage of this resource to make their protocol details widely available.

While we agree that this information is important, we are hesitant to include too many layers of structured fields which might deter users from engaging with the minimum information standards. Further, since the goal is to enable search and discovery of related datasets, we think that focusing on categorical information provides the right balance.

Having said that, we agree that some parts of the schema were under-specified and have added a way to provide identifiers (such as strain and cell line) in a structured format and added this information to the examples.

Reviewer #2: This study reports the development of minimum information and guidelines for reporting a multiplexed assay of variant effect. Overall, the challenges to be addressed are properly identified, and the method reported is the state-of-the-art.

However, more details appear to be needed for this work. First of all, It is hard to know from current paper what is really "minimal" in terms of the minimal information.

We have added detailed documentation to the GitHub documentation describing the standard and providing further guidance for users who want to apply it to their data. As part of this, we also describe which fields are required by the standard as currently implemented.

The paper emphasizes the usage of ontologies such as OBI and CLO, which is great. However, more details would be better. For example, it may be possible to provide a full list of ontologies to be used.

We thank the reviewer for this suggestion. We have added a list of ontologies and identifiers (including links to the resources) used by the schema to the documentation on GitHub and also included this information in the list of controlled vocabulary terms.

We also updated our examples to include the use of CLO, OBI, and NCBI TaxID terms.

it appears difficult to evaluate the quality and value of the minimal information standard. Ideally, the authors need to provide some solid example to demonstrate the usage of the minimal information standard. Currently, Figure 1 provides some simple demo. However, it is hard to evaluate from this example.

We agree that additional structured examples will make it much easier for researchers to understand how to use the guidelines. In addition to the previous example (from PMID 36400770), we have added examples for two additional popular and distinct experimental methodologies to GitHub, including VAMP-seq (from PMID 29785012) and saturation genome editing (from PMID 30209399). We have also revamped Figure 1 in the paper to highlight these three examples and hopefully make usage of the standards more clear.

**Second round of review**

**Reviewer 1**

The authors have provided thoughtful responses to my comments in the first round of review. These resulted in some minor textual edits and a major redesign of Figure 1. The new Figure 1 is much improved. The most substantial changes are to the Github repository. This has been completely updated, and now provides comprehensive guidance for preparing the minimum information standard. This guidance is both practical (how to approach preparing the document) and technical (specification of the terms in the controlled vocabulary). The authors have done an excellent job in creating this online resource, and the detailed guidance will likely aid greatly in adoption. Although the authors did not choose to incorporate all of my suggestions from the previous round of review, this revision has addressed all of the concerns that I indicated as essential, and I am in full support for publication of this revised version.

Minor comments:

On Github, Readme.md “and modifying” grammar.

**Reviewer 2**

The authors have properly addressed my comments, and so I would like to accept this paper.
